# Electrocatalytic miRNA Detection Using Cobalt Porphyrin-Modified Reduced Graphene Oxide

**DOI:** 10.3390/s140609984

**Published:** 2014-06-06

**Authors:** Camille De Souza, Samia Zrig, Dengjun Wang, Minh-Chau Pham, Benoit Piro

**Affiliations:** University Paris Diderot, Sorbonne Paris Cité, ITODYS, UMR 7086 CNRS, 15 rue J-A de Baïf, 75205 Paris Cedex 13, France; E-Mails: ca.desouza@yahoo.fr (C.D.S.); samia.zrig@univ-paris-diderot.fr (S.Z.); dengjun.wang@univ-paris-diderot.fr (D.W.); mcpham@univ-paris-diderot.fr (M.-C.P.)

**Keywords:** reduced graphene oxide, microRNA, porphyrin, hybridization, reagentless detection

## Abstract

Metalated porphyrins have been described to bind nucleic acids. Additionally, cobalt porphyrins present catalytic properties towards oxygen reduction. In this work, a carboxylic acid-functionalized cobalt porphyrin was physisorbed on reduced graphene oxide, then immobilized on glassy carbon electrodes. The carboxylic groups were used to covalently graft amino-terminated oligonucleotide probes which are complementary to a short microRNA target. It was shown that the catalytic oxygen electroreduction on cobalt porphyrin increases upon hybridization of miRNA strand (“signal-on” response). Current changes are amplified compared to non-catalytic amperometric system. Apart from oxygen, no added reagent is necessary. A limit of detection in the sub-nanomolar range was reached. This approach has never been described in the literature.

## 1. Introduction

Recently, a new class of short non-coding ribonucleic acids (RNA) was found to play an important role in translation regulation and RNA degradation. Called microRNA, miRNA or miR, they are 18–30 bases long, and more than a thousand were identified up to now. MicroRNA can be considered as biomarkers whose detection, even at the trace level, may allow early diagnostics of cancer [1–4], but also of other affections such as diabetes or cardiac diseases. For example, miR-29b-1 was detected at high level in serum of patients having bone disease.

Up to now, detection and quantification of miRNA relied on classical techniques of molecular biology such as Northern Blot, qRT-PCR or even optical DNA chips based on fluorescence labeling or SPR [5–8]. These well-recognized methods all have limitations, however. Indeed, they need use of added markers or reactants (fluorescent or redox), several detection steps and are time consuming. These techniques are therefore difficult to use for rapid detection and without qualified operators.

Nowadays, to gain time at lower cost is essential. Biosensors can provide these advantages; they are devices able to detect and quantify “target” molecules as straightforwardly as possible, through a directly measurable electrical signal. They are based on the association of a molecular recognition element with a transducer; the first react or bind specifically to the analyte of interest while the second converts this molecular recognition into a measurable signal. For a DNA biosensor, the principle is based on the specific association (hybridization) of nucleobases (A-T and G-C) to form a duplex from the probe and target sequences. The same principle can be used to detect ribonucleic acids (RNA or miRNA), except that T is replaced by U to form A-U association. RNA.DNA hybridization leads to hybrids whose stability (*i.e.*, the melting temperature) is significantly higher than DNA.DNA duplexes [9].

Hybridization of miRNA may be transduced by several techniques, most of them being optical [10,11]. Rather than that, in this article, we were interested in an electrochemical transduction, and more precisely a direct and reagentless one [12]. We evidenced the modulation of the electrocatalytic properties of a cobalt tetra(4-carboxyphenyl)porphyrin, π-stacked on reduced graphene oxide, when miRNA hybridized with DNA probes covalently coupled to the carboxyl group carried by the porphyrin.

Porphyrins are tetrapyrrolic aromatic macrocycles with an 18 electron π-conjugated system. They present a coordination site for various transition metals at different oxidation states, so that the electronic properties of porphyrins depend on the coordinated metal, but also depend on its substituents [13]. They play important roles for catalytic oxidation or reduction reactions of various molecules such as CO_2_ or O_2_ [14–17]. For example, cobalt tetra(4-carboxyphenyl)porphyrin (CoTCPP) is known to catalyze oxygen reduction with a 2- or 4-electron transfer, through an outer sphere mechanism [18,19]. It has been also described that some porphyrins, including cobalt porphyrins, may interact with nucleic acids with binding constants in the range of 10^5^−10^6^ M^−1^ [20–22] and that this binding influences optical properties of the porphyrins. However changes in their electrochemical properties, more particularly their catalytic properties towards oxygen reduction, were not investigated.

Examples of porphyrin self-assembly on various substrates are reported in the literature [23]. Porphyrins can irreversibly adsorb through π-π interactions on graphite [24], carbon nanotubes [25,26] and graphene [27–30]. Following this approach, we modified reduced graphene oxide (RGO) with CoTCPP, which we deposited on glassy carbon (GC) electrodes afterwards. RGO brings excellent electrical conductivity, high specific surface area and porous character which allow to play on diffusion kinetics, while CoTCPP brings its intrinsic electrocatalytic properties. CoTCPP presents four phenyl-carboxylic substituents on meso positions 5, 10, 15 and 20; these carboxylic groups were used for covalent coupling of an amino-modified oligonucleotide probe, pDNA-29b-1, which is complementary to miR-29b-1, a microRNA related to human osteoclastic cell differentiation and bone diseases [31]. pDNA-29b-1.miR29b-1 hybridization was monitored in aerated phosphate buffer by recording the oxygen reduction peak using square wave voltammetry between 0 V and ‐0.4 V *vs.* SCE.

## 2. Experimental Section

*Chemicals.* Reagents (hydrochloric acid HCl, colbalt acetate (CH_3_COO_2_)Co) and solvents (acetonitrile ACN, ethanol EtOH, dimethylformamide DMF) were PA grade. Hydrazine (65% in water), phosphate buffer saline tablets (PBS, 137 mM NaCl; 2.7 mM KCl; 8.1 mM Na_2_HPO_4_; 1.47 mM KH_2_PO_4_, pH 7.4) and perchlorate (LiClO_4_) were purchased from Sigma-Aldrich (St. Louis, MO, USA). 1-(3-Dimethylaminopropyl)-3-ethylcarbodiimide hydrochloride (EDC, purity 98%) and *N*-hydroxysuccinimide (NHS, purity 98%) were from Alfa Aesar (Ward Hill, MA, USA). Single-layer graphene oxide (size 1–5 μm; thickness 0.8–1.2 nm) was purchased from ACS Material LLC (Medford, MA, USA), synthesized using the modified Hummer's method. Aqueous solutions were made with ultrapure (18 MΩ·cm) water. Glassy carbon working electrodes (3 mm diameter, S = 0.07 cm^2^) were purchased from BASi (West Lafayette, IN, USA). All oligonucleotides were purchased from Eurogentec (Liege, Belgium) and are detailed in Table 1. miR-29b-1 is a miRNA related to bone diseases, whereas miR-141, related to prostate cancer, was used as a non-specific target. Tetra(4-carboxyphenyl)porphyrine (TCPP) was metalated according to procedure described below.

*Preparation of RGO-Modified Electrodes.* Reduced graphene oxide (RGO) was obtained from graphene oxide (GO) following a protocol described in the literature [32]. Graphene oxide (17 mg) was dispersed in ultrapure water (17 mL) under ultrasonication for 30 min. After addition of hydrazine (160 μL, 65% in water), the mixture was heated at 80 °C under magnetic stirring for 12 h. The resulting slurry was cooled down to room temperature, filtered and washed with ultrapure water to obtain reduced graphene oxide (RGO) as a black precipitate, which was dried under vacuum before use.

*Synthesis of Metalated Cobalt Tetra(4-carboxyphenyl)Porphyrin (TCPP)*. In a glass tube adapted to microwave, cobalt acetate (36 mg, 3 eq) was added to a solution of TCPP (25 mg, 1 eq) in DMF (5 mL). The reaction medium was microwaved during 20 min at 120 °C (800 W), then the solvent was evaporated under vacuum. The solid was recrystallized in chlorhydric acid. The metalated porphyrin was filtered and washed three times in water, then dried under vacuum.

*Probe Grafting and miRNA Hybridization*. NH_2_-modified DNA probes (pDNA-29b-1) were covalently grafted on CoTCPP/RGO/GCE in 0.1 M MES buffer containing 150 mM EDC + 300 mM NHS. The reaction was carried out overnight at 37 °C. Electrodes were then washed with water and PBS and incubated in PBS at 37 °C for 1 h to release physisorbed DNA probes.

*Methods and Apparatus*. For electrochemical experiments, a conventional one-compartment, three-electrode cell was used with a VMP3 potentiostat (Bio-Logic, Claix, France), the data being collected by EC-lab^®^ software from Bio-Logic. To prepare the working electrodes, their surface were polished with 0.3 μm alumina slurry on microfiber pads for 1 min. The residual alumina particles were removed by sonication in distilled water then acetonitrile for 30 s, respectively. The electrodes were air-dried before use. The counter electrode was a platinum grid and the reference electrode a commercial saturated calomel electrode—SCE (Metrohm, Villebon, Swisserland). The electrolytic solution was argon-saturated PBS, or aerated PBS for oxygen reduction experiments. Square wave voltammetry (SWV) scans were repeated until complete stabilization of the electrochemical signal (*i.e.*, no difference observed between two successive SWV scans). The metalation reaction was performed with a microwave synthesis reactor Anton Paar Monowave 300 (Graz, Austria).

## 3. Results and Discussion

### 3.1. Electrochemical Characterization of O_2_ Electroreduction on CoTCPP-Modified Electrodes

**Figure 1.**
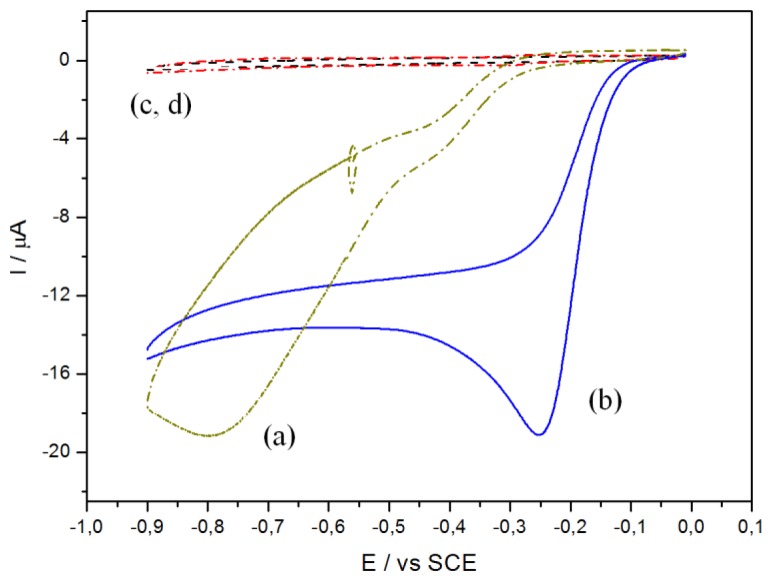
Cyclic voltammograms of a non-functionalized GC electrode (curve **a**) and a GC electrode functionalized by drop-casting (10^−4^ M in 10 μL H_2_O) of CoTCPP (curve **b**) in oxygen-saturated PBS, or in deaerated PBS (curves **c**,**d**), respectively. Medium: PBS; v = 50 mV s^−1^; E *vs.* SCE; S = 0.07 cm^2^.

Figure 1 presents the electrochemical characterization of O_2_ electroreduction. Curve (a) presents the typical behavior of O_2_ reduction on GC, with a reduction starting below ‐0.2 V and a peak maximum situated at *ca.* ‐0.8 V *vs.* SCE. Conversely, curve (b) shows that O_2_ reduction on CoTCPP-modified GC electrodes starts at higher potential (*ca.* 0 V), presenting a steeper slope and a peak maximum at *ca.* ‐0.25 V *vs.* SCE. Without oxygen, on bare GC or CoTCPP/GC electrodes (curves c and d, respectively), the reduction current is not significant (approximately 30-fold smaller). CoTCPP, in deaerated medium, does not present any significant electroactivity (not shown), as described in the literature.

### 3.2. RGO-Modified Electrodes

**Figure 2.**
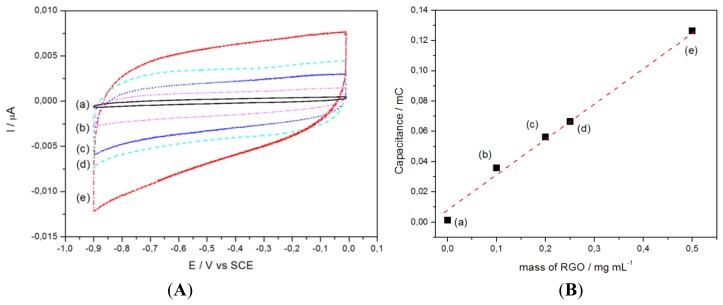
(**A**) CVs of a RGO-modified GC electrode, for various quantities of drop-casted RGO: (a) 0 μg cm^−2^; (b) 100 μg cm^−2^; (c) 200 μg cm^−2^; (d) 250 μg cm^−2^; (e) 500 μg cm^−2^. (**B**) Capacitive charge derived from CVs of Figure 2A. Medium: deaerated PBS; v = 50 mV s^−1^, S = 0.07 cm^2^.

Reduced graphene oxide (RGO) was used in order to immobilize CoTCPP through strong π-π interactions [27–30], and to increase the specific surface area of GC electrodes. First of all, various quantities of RGO were drop-casted on electrodes, and the electrochemical capacitance measured by cyclic voltammetry (Figure 2A).

Figure 2B shows the capacitive charge as a function of the RGO quantity immobilized on the electrodes. The areal capacitance is approximately 300 F g^−1^ cm^−2^.

### 3.3. RGO: CoTCPP-Modified Electrodes

RGO was first modified with CoTCPP by mixing RGO and CoTCPP in a 1:2 water-acetonitrile (vol/vol) mixture under magnetic stirring during 72 h, then isolated by centrifugation and washed first with ethanol then with distillated water. The best result was obtained with 200 μg mL^−1^ RGO and 1.8 × 10^−4^ M CoTCPP, for which the resulting CoTCPP-modified RGO can form a stable suspension in water up to 1.17 mg mL^−1^. These conditions were used for the following experiments.

**Figure 3.**
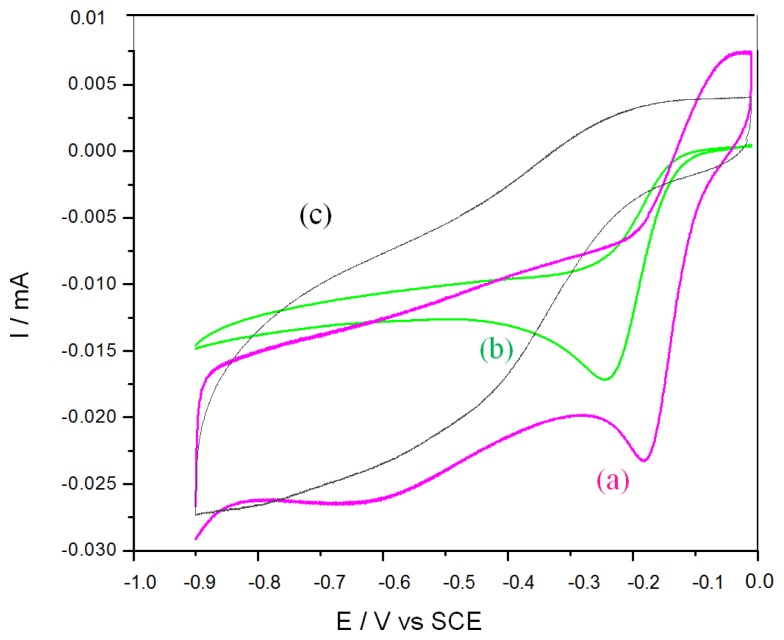
Oxygen electroreduction on (**a**) CoTCPP/RGO, (**b**) CoTCPP and (**c**) RGO-modified glassy carbon electrodes in aerated PBS. T = 25 °C, S = 0.07 cm^2^, v = 50 mV s^−1^. Electrodes were modified by drop-casting of 10 μL of a suspension of 200 μg mL^−1^ CoTCPP/RGO in ultrapure water.

A suspension of 200 μg mL^−1^ CoTCPP/RGO in ultrapure water (10 μL) was drop-casted on GC electrodes, then let to dry 24 h before use. Figure 3 shows cyclic voltammograms of CoTCPP/RGO-modified, CoTCPP-modified and RGO-modified GC electrodes, under oxygenated or deoxygenated conditions. As previously shown in Figure 1, on bare GC electrode, oxygen electroreduction occurs at low potentials (curve a). On a RGO-modified electrode, reduction starts at potentials slightly higher (Figure 3, curve c); however, the peak maximum occurs below ‐0.8 V. On a CoTCPP-modified electrode (Figure 3, curve b), the electroreduction peak occurs at ‐0.26 V. On a CoTCPP/RGO-modified electrode (Figure 3, curve a), the electroreduction wave is shifted 70 mV more positive than without RGO, with a peak current slightly higher. In these experimental conditions, the peak current is limited by oxygen diffusion.

### 3.4. miRNA Detection

To graft pDNA-29b-1 probes, CoTCPP/RGO-modified GC electrodes were dipped into 500 μL of a solution containing 150 mM EDC + 300 mM NHS, at 37 °C for 2 h. After that, the electrodes were washed with ultrapure water and immersed in 500 μL of H_2_O + 10^−7^ M pDNA-29b-1 for 2 h at 37 °C, then washed and rinsed with PBS during 45 min at 37 °C under stirring, then with ultrapure water. Hybridization solutions containing target miRNA in PBS (from 10^−11^ M up to 10^−9^ M) were prepared and heated above the melting temperature of the duplex for 5 min. pDNA-29b-1/CoTCPP/RGO electrodes were dipped into this solution and then kept at the hybridization temperature for 2 h, then washed with PBS at 50 °C. Immediately, SWV was used to characterize hybridization. The main oxygen reduction peak current (situated slightly above ‐0.2 V *vs.* SCE) was used as the transduction signal.

**Figure 4.**
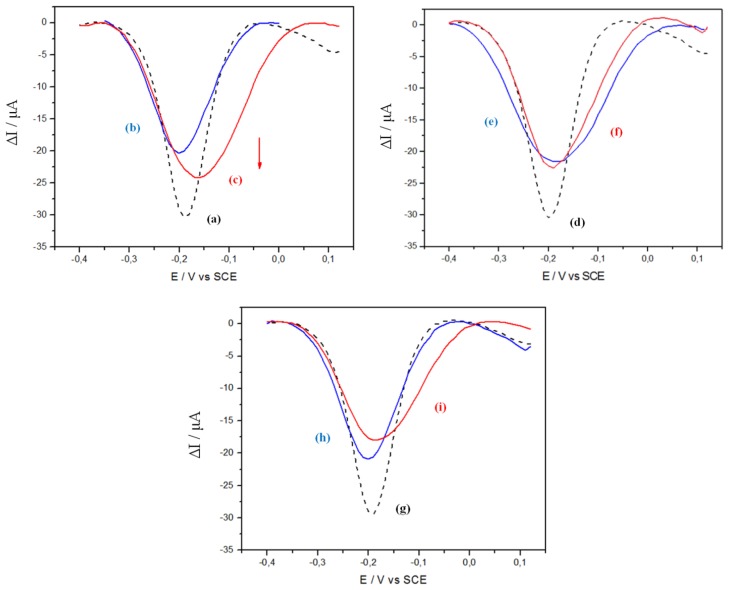
Square wave voltammetry in PBS of a CoTCPP/RGO electrode before grafting of pDNA-29b-1 probe (**a**,**d**,**g**), after grafting of the pDNA-29b-1 probe (**b**,**e**,**h**) and after hybridization with miR-29b-1target (**c**), PBS only (**f**) and miR-141 non-complementary target (**i**); O_2_ saturated medium; target concentration of 10^−9^ M (when present); v = 100 mV s^−1^; S = 0.07 cm^2^.

As shown on Figure 4, curve b, pDNA-29b-1 probe grafting leads to a relative current decrease of −30% compared to curve a (no grafted probe), while hybridization with the complementary miR-29b-1 strand (10^−9^ M) leads to a +20% increase (curve c) compared to curve b. Under the same experimental conditions, but for incubation without any miR target (curve f), the current change is not significant, while one can observe a slight current decrease (−10%) for incubation with a non-complementary target (miR-141, curve i). These experiments, replicated at least three times, gave the following averaged current changes, for a concentration of miR target (when present) of 10^−9^ M: grafting (−25 ± 8)%, hybridization (+18 ± 8)%, blank (0 ± 2)%, non-complementary (−10 ± 3)%.

One can make the hypothesis that the conformational change [33] induced by the transition from single strand pDNA-29b-1 probe to double-stranded pDNA-29b-1. miR29b-1 hybrid influences the reduction current. This could be due to changes in weak bond interactions between the DNA strands and the porphyrin [20–22], or changes in the diffusion kinetic of oxygen at the graphene interface.

**Figure 5.**
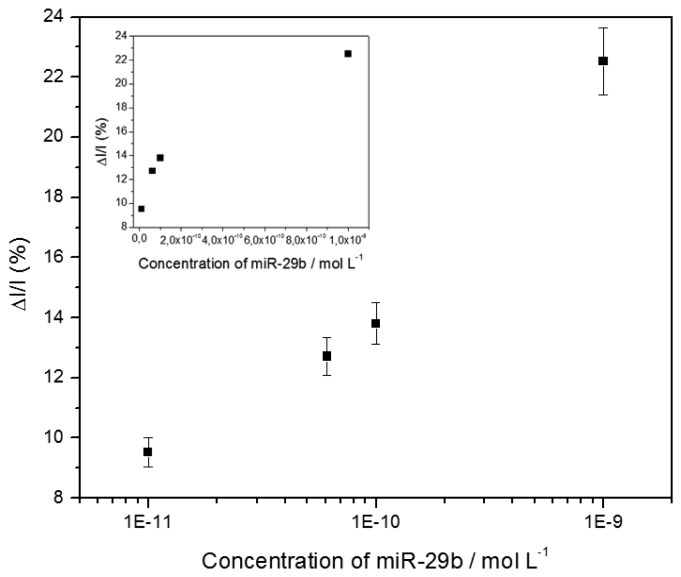
Relative SWV peak current changes recorded after hybridization with the complementary target miR-29b-1 at various concentrations between 10^−11^ M and 10^−9^ M. Same experimental conditions as in Figure 4. Inset: same data, plotted in decimal scale.

Various target concentrations were investigated, from 10^−11^ M to 10^−9^ M. The calibration curve was constructed from the relative current change, expressed as a percentage (%*ΔI_peak_/I_peak_*), before and after hybridization, using the following Equation (1):

(1) $$\%\frac{\Delta I_{\text{peak}}}{I_{\text{peak}}}=\frac{I_{\text{peak},\text{Hyb}.}-I_{\text{peak},\text{probe}.}}{I_{\text{peak},\text{probe}}}\times 100$$

where *I_Peak_*,*_Probe_* and *I_Peak_*,*_Hyb_* are the currents corresponding to the SWV reduction peak at ‐0.2 V before and after hybridization, respectively (Figure 5).

## 4. Conclusions

We have elaborated CoTCPP/RGO-modified electrodes by reducing graphene oxide then functionalizing it by CoTCPP through π-π interactions. These electrodes combine the high electrical conductivity and specific area of RGO with the electrocatalytic properties of cobalt porphyrin towards oxygen electroreduction, in neutral saline solution (PBS). CoTCPP carries carboxylic groups which were used to covalently graft oligonucleotide probes through peptide bonds. In addition, CoTCPP may interact with nucleic acids through weak bonds. Hybridization of this probe with short miRNA targets leads to a change in conformation of these DNA strands, from a random coil structure to a well-organized double-strand. This molecular reorganization may influence the electrocatalytic behavior of CoTCPP towards oxygen reduction through two different ways. First, weak interactions between CoTCPP and the nucleic acids may be broken upon hybridization; secondly, the diffusion of oxygen, which was lowered due to the randomly coiled probes, may be restored upon hybridization. These two phenomena may participate to the current increase which was recorded. To the best of our knowledge, it is the first reported attempt to transduce miRNA. DNA hybridization through an electrocatalytic system based on porphyrin and oxygen reduction. This catalytic approach, conjugated to the use of reduced graphene oxide, allows high current densities despite very low quantity of electroactive material. Detection of miRNA was taken as a possible application example because they are promising disease response biomarkers. For application in concrete cases, optimizations will be necessary (decrease of the detection limit, analysis in complex media, sensitivity to single-nucleotide mismatch).

## Figures and Tables

**Table 1.** List of DNA and miRNA sequences.

**ODN Name**	**Function**	**Bases**	**Sequences**
pDNA-29b-1	Probe DNA	23	5′ NH_2_-AACACTGATTTCAAATGGTGCTA 3′
miR-141	Target RNA	22	3′ GGUAGAAAUGGUCUGUCACAAU 5′
miR-29b-1	Target RNA	23	3′ UUGUGACUAAAGUUUACCACGAU 5′
